# 4-(Prop-2-yn-1-yl­oxy)benzene-1,2-dicarbonitrile

**DOI:** 10.1107/S1600536812028309

**Published:** 2012-06-30

**Authors:** Yee Jan Chin, Ai Ling Tan, Franz L. Wimmer, Aminul Huq Mirza, David J. Young, Seik Weng Ng, Edward R. T. Tiekink

**Affiliations:** a Faculty of Science, Universiti Brunei Darussalam, Jalan Tungku Link BE 1410, Negara, Brunei Darussalam; b Department of Chemistry, University of Malaya, 50603 Kuala Lumpur, Malaysia; c Chemistry Department and Faculty of Science, King Abdulaziz University, PO Box 80203 Jeddah, Saudi Arabia

## Abstract

In the title compound, C_11_H_6_N_2_O, the complete mol­ecule is generated by the application of crystallographic twofold symmetry (the mol­ecule is disordered about this axis). The prop-2-yn-1-yl residue is slightly twisted out of the plane of the benzene ring [C—O—C—C torsion angle = 173.1 (3)°] and is orientated away from the nitrile substituents. In the crystal, supra­molecular chains along the *a* axis, arising from C—H⋯N inter­actions, are connected into stacks along the *c* axis by π–π inter­actions between the benzene rings [centroid–centroid distance = 3.6978 (6) Å = length of the *c* axis].

## Related literature

For the solubilization and some applications of phthanocyanine dyes, see: Jiang *et al.* (2011 ▶); Sleven *et al.* (2001 ▶). For the synthesis of substituted phthalonitriles, see: Wöhrle *et al.* (1993 ▶); Wu *et al.* (1998 ▶); Li & Lieberman (2001 ▶); Sleven *et al.* (2001 ▶); Li *et al.* (2008 ▶); Seven *et al.* (2009 ▶); Foo *et al.* (2012 ▶).
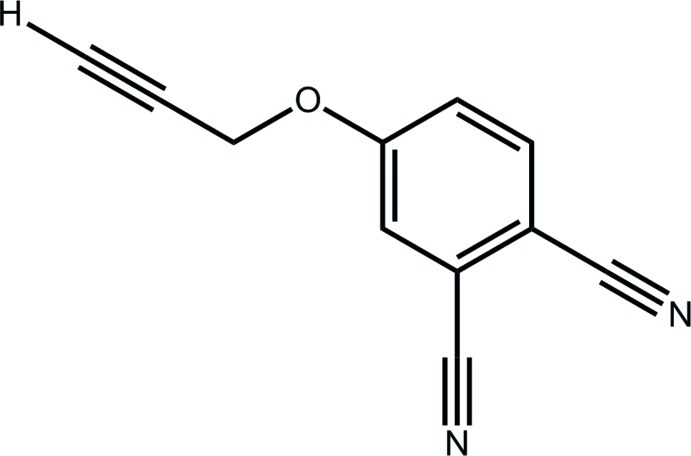


## Experimental

### 

#### Crystal data

C_11_H_6_N_2_O
*M* *_r_* = 182.18
Monoclinic, 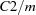

*a* = 11.4809 (9) Å
*b* = 22.2091 (16) Å
*c* = 3.6978 (6) Å
β = 91.304 (10)°
*V* = 942.62 (18) Å^3^
*Z* = 4
Mo *K*α radiation
μ = 0.09 mm^−1^
*T* = 100 K
0.15 × 0.05 × 0.05 mm

#### Data collection

Agilent SuperNova Dual diffractometer with an Atlas detector
Absorption correction: multi-scan (*CrysAlis PRO*; Agilent, 2012 ▶) *T*_min_ = 0.792, *T*_max_ = 1.000
3258 measured reflections
1114 independent reflections
840 reflections with *I* > 2σ(*I*)
*R*_int_ = 0.038

#### Refinement

*R*[*F* ^2^ > 2σ(*F* ^2^)] = 0.059
*wR*(*F* ^2^) = 0.163
*S* = 1.08
1114 reflections
86 parameters
12 restraints
H atoms treated by a mixture of independent and constrained refinement
Δρ_max_ = 0.41 e Å^−3^
Δρ_min_ = −0.28 e Å^−3^

### 

Data collection: *CrysAlis PRO* (Agilent, 2012 ▶); cell refinement: *CrysAlis PRO*; data reduction: *CrysAlis PRO*; program(s) used to solve structure: *SHELXS97* (Sheldrick, 2008 ▶); program(s) used to refine structure: *SHELXL97* (Sheldrick, 2008 ▶); molecular graphics: *ORTEP-3* (Farrugia, 1997 ▶) and *DIAMOND* (Brandenburg, 2006 ▶); software used to prepare material for publication: *publCIF* (Westrip, 2010 ▶).

## Supplementary Material

Crystal structure: contains datablock(s) global, I. DOI: 10.1107/S1600536812028309/hb6862sup1.cif

Structure factors: contains datablock(s) I. DOI: 10.1107/S1600536812028309/hb6862Isup2.hkl

Supplementary material file. DOI: 10.1107/S1600536812028309/hb6862Isup3.cml

Additional supplementary materials:  crystallographic information; 3D view; checkCIF report

## Figures and Tables

**Table 1:** Hydrogen-bond geometry (Å, °)

*D*—H⋯*A*	*D*—H	H⋯*A*	*D*⋯*A*	*D*—H⋯*A*
C3—H3⋯N1^i^	0.95	2.62	3.509 (3)	155

Symmetry code: (i) 
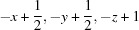
.

**Fig. 1.**
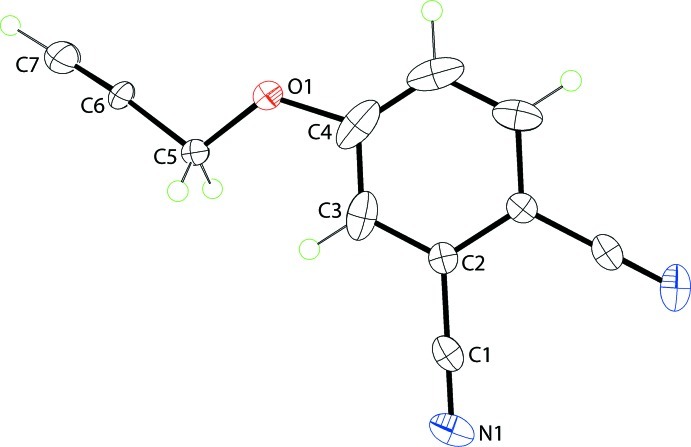
The molecular structure of (I) showing displacement ellipsoids at the 50% probability level. The molecule is disordered about the 2-fold axis - only one orientation is shown.

**Fig. 2.**
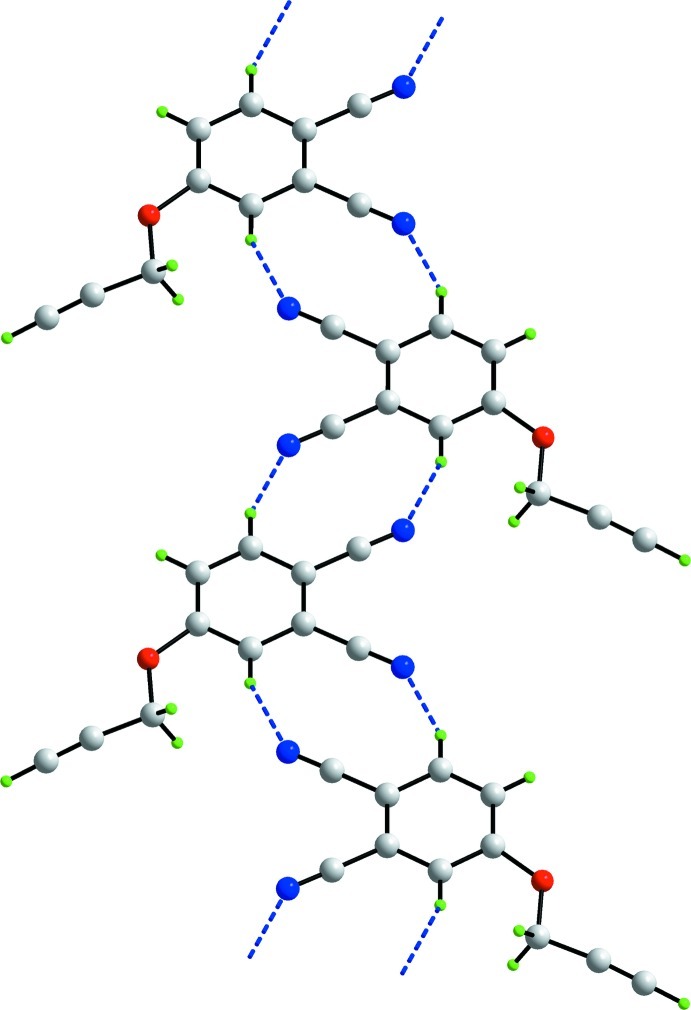
A view of the supramolecular chain along the a axis in (I). The C—H···N and interactions are shown as blue dashed lines.

**Fig. 3.**
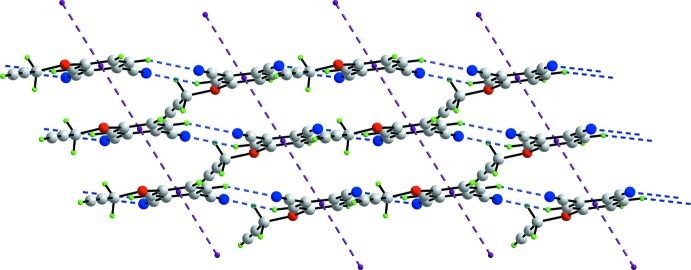
A view of the supramolecular layer in the ac plane in (I). The C—H···O and π—π interactions are shown as blue and purple dashed lines, respectively.

## supplementary crystallographic information

### Comment

Phthalocyanine dyes can be made soluble in water and organic solvents by addition of suitable alkoxy or aryloxy groups (Jiang *et al.*, 2011; Sleven *et al.*, 2001). This is most easily achieved from the correspondingly substituted phthalonitriles which, in turn, are either prepared by Sandmeyer reaction of alkyl or alkoxy functionalized dihalobenzenes (Li & Lieberman, 2001; Sleven *et al.*, 2001) or aryloxy / alkoxy displacement of the corresponding halophthalonitrile (Wöhrle *et al*., 1993; Li *et al.*, 2008; Foo *et al.*, 2012) or 4-nitrophthalonitrile (Wu *et al.*, 1998; Seven *et al.*, 2009). The latter method is most suitable for preparing 4-alkoxyphthalonitriles and was used for preparing the title compound, 4-(prop-2-ylnyloxy)phthalonitrile (I).

In (I), Fig. 1, the complete molecule is generated by the application of 2-fold symmetry; the molecule is disordered about this axis. The O1 and C1 atoms lie -0.067 (3) and 0.059 (2) Å out of the plane through the benzene ring, respectively. The prop-2-yn-1-yl is twisted out of the plane of the benzene ring as seen in the value of the C4—O1—C5—C6 torsion angle of 173.1 (3)° and is orientated in the opposite direction to the nitrile substituents.

In the crystal packing, supramolecular chains along the *a* axis feature owing to C—H···N interactions, Table 1, and 10-membered {···HC_3_N}_2_ synthons, Fig. 2. Chains are connected into stacks along the *c* axis by π—π interactions between the benzene rings [inter-centroid distance = 3.6978 (6) Å = length of the *c* axis]. The layers inter-digitate along the *b* axis with no specific intermolecular interactions between them.

### Experimental

The title compound was prepared by modification of literature procedures (Wu *et al.*, 1998; Seven *et al.*, 2009). Under a nitrogen atmosphere, anhydrous potassium carbonate (1.12 g, 8.1 mmol) was added in two portions at 1 h intervals to a solution of propargyl alcohol (1.5 ml, 26.0 mmol) and 4-nitrophthalonitrile (0.70 g, 4.04 mmol) in dry *N*,*N*-dimethylformamide (7 ml). After 96 h, the crude reaction mixture was poured into water (140 ml). The green precipitate was collected by vacuum filtration, washed with water and dried. The crude product was purified by silica gel column chromatography using dichloromethane as eluent to provide 0.4 g (63.9%) of a faintly coloured solid that was recrystallized from CH_2_Cl_2_ / hexane as colourless prisms. Melting point = 383 K. IR ν/cm^-1^: 3287, 3119, 3077, 2231, 2135, 1596, 1494, 1321, 1260. ^1^H NMR 400 MHz (CDCl_3_) δ: 7.75 (1*H*, d), 7.35 (1*H*, s), 7.28 (1*H*, d), 4.80 (2*H*, s), 2.62 (1*H*, s).

### Refinement

With the exception of the acetylenic H-atom which was refined freely, carbon-bound H-atoms were placed in calculated positions [C—H = 0.95–0.99 Å, *U*_iso_(H) = 1.2*U*_eq_(C)] and were included in the refinement in the riding model approximation. The molecule is disordered over a 2-fold rotation axis in an exact 1:1 ratio. The anisotropic displacement parameters of the O1 and C4 atoms were tightly restrained to be nearly isotropic.

### Crystal data

**Table:**

C_11_H_6_N_2_O	*F*(000) = 376
*M**_r_* = 182.18	*D*_x_ = 1.284 Mg m^−^^3^
Monoclinic, *C*2/*m*	Mo *K*α radiation, λ = 0.71073 Å
Hall symbol: -C 2y	Cell parameters from 938 reflections
*a* = 11.4809 (9) Å	θ = 3.3–27.5°
*b* = 22.2091 (16) Å	µ = 0.09 mm^−^^1^
*c* = 3.6978 (6) Å	*T* = 100 K
β = 91.304 (10)°	Prism, colourless
*V* = 942.62 (18) Å^3^	0.15 × 0.05 × 0.05 mm
*Z* = 4	

### Data collection

**Table:**

Agilent SuperNova Dual diffractometer with an Atlas detector	1114 independent reflections
Radiation source: fine-focus sealed tube	840 reflections with *I* > 2σ(*I*)
Mirror monochromator	*R*_int_ = 0.038
Detector resolution: 10.4041 pixels mm^-1^	θ_max_ = 27.6°, θ_min_ = 3.3°
ω scan	*h* = −14→14
Absorption correction: multi-scan (*CrysAlis PRO*; Agilent, 2012)	*k* = −26→28
*T*_min_ = 0.792, *T*_max_ = 1.000	*l* = −4→4
3258 measured reflections	

### Refinement

**Table:**

Refinement on *F*^2^	Primary atom site location: structure-invariant direct methods
Least-squares matrix: full	Secondary atom site location: difference Fourier map
*R*[*F*^2^ > 2σ(*F*^2^)] = 0.059	Hydrogen site location: inferred from neighbouring sites
*wR*(*F*^2^) = 0.163	H atoms treated by a mixture of independent and constrained refinement
*S* = 1.08	*w* = 1/[σ^2^(*F*_o_^2^) + (0.0718*P*)^2^ + 0.8054*P*] where *P* = (*F*_o_^2^ + 2*F*_c_^2^)/3
1114 reflections	(Δ/σ)_max_ < 0.001
86 parameters	Δρ_max_ = 0.41 e Å^−^^3^
12 restraints	Δρ_min_ = −0.28 e Å^−^^3^

### Special details

**Table:**

Geometry. All e.s.d.'s (except the e.s.d. in the dihedral angle between two l.s. planes) are estimated using the full covariance matrix. The cell e.s.d.'s are taken into account individually in the estimation of e.s.d.'s in distances, angles and torsion angles; correlations between e.s.d.'s in cell parameters are only used when they are defined by crystal symmetry. An approximate (isotropic) treatment of cell e.s.d.'s is used for estimating e.s.d.'s involving l.s. planes.
Refinement. Refinement of *F*^2^ against ALL reflections. The weighted *R*-factor *wR* and goodness of fit *S* are based on *F*^2^, conventional *R*-factors *R* are based on *F*, with *F* set to zero for negative *F*^2^. The threshold expression of *F*^2^ > σ(*F*^2^) is used only for calculating *R*-factors(gt) *etc*. and is not relevant to the choice of reflections for refinement. *R*-factors based on *F*^2^ are statistically about twice as large as those based on *F*, and *R*- factors based on ALL data will be even larger.

### Fractional atomic coordinates and isotropic or equivalent isotropic displacement parameters (Å^2^)

**Table:**

	*x*	*y*	*z*	*U*_iso_*/*U*_eq_	Occ. (<1)
O1	0.1308 (2)	0.45076 (11)	0.1768 (8)	0.0273 (7)	0.50
N1	0.15188 (16)	0.19174 (9)	0.2675 (6)	0.0376 (6)	
C1	0.10982 (16)	0.23658 (9)	0.1829 (6)	0.0260 (5)	
C2	0.05529 (16)	0.29272 (8)	0.0857 (5)	0.0219 (5)	
C3	0.1103 (2)	0.34676 (10)	0.1663 (6)	0.0349 (6)	
H3	0.1853	0.3469	0.2801	0.042*	
C4	0.0555 (3)	0.40028 (10)	0.0803 (7)	0.0446 (7)	
H4	0.0936	0.4374	0.1309	0.054*	0.50
C5	0.2499 (3)	0.44609 (17)	0.3108 (13)	0.0274 (10)	0.50
H5A	0.2962	0.4211	0.1458	0.033*	0.50
H5B	0.2522	0.4272	0.5535	0.033*	0.50
C6	0.2980 (3)	0.5082 (7)	0.3303 (12)	0.030 (3)	0.50
C7	0.3440 (4)	0.5544 (2)	0.3459 (17)	0.0463 (14)	0.50
H7	0.386 (5)	0.595 (3)	0.363 (16)	0.054 (16)*	0.50

### Atomic displacement parameters (Å^2^)

**Table:**

	*U*^11^	*U*^22^	*U*^33^	*U*^12^	*U*^13^	*U*^23^
O1	0.0258 (13)	0.0187 (12)	0.0370 (15)	0.0001 (10)	−0.0069 (11)	−0.0008 (11)
N1	0.0332 (10)	0.0410 (11)	0.0383 (12)	0.0126 (9)	−0.0029 (8)	0.0074 (9)
C1	0.0191 (9)	0.0345 (12)	0.0242 (11)	−0.0003 (8)	−0.0019 (8)	0.0004 (8)
C2	0.0206 (10)	0.0234 (10)	0.0217 (10)	−0.0009 (7)	−0.0001 (8)	−0.0007 (7)
C3	0.0401 (12)	0.0399 (13)	0.0251 (12)	−0.0182 (10)	0.0071 (9)	−0.0077 (9)
C4	0.0728 (15)	0.0262 (10)	0.0355 (13)	−0.0161 (10)	0.0172 (11)	−0.0063 (9)
C5	0.0220 (19)	0.0167 (19)	0.043 (3)	0.0005 (17)	−0.0104 (17)	−0.0001 (16)
C6	0.0262 (16)	0.013 (9)	0.051 (2)	−0.004 (2)	−0.0130 (15)	−0.001 (2)
C7	0.033 (3)	0.027 (2)	0.078 (4)	0.001 (2)	−0.019 (2)	−0.001 (2)

### Geometric parameters (Å, º)

**Table:**

O1—C5	1.447 (5)	C4—C4^i^	1.393 (6)
O1—C4	1.455 (3)	C4—H4	0.9500
N1—C1	1.147 (3)	C5—C6	1.487 (14)
C1—C2	1.437 (3)	C5—H5A	0.9900
C2—C3	1.385 (3)	C5—H5B	0.9900
C2—C2^i^	1.406 (4)	C6—C7	1.155 (16)
C3—C4	1.379 (3)	C7—H7	1.02 (6)
C3—H3	0.9500		
			
C5—O1—C4	125.5 (3)	C3—C4—H4	119.8
N1—C1—C2	178.4 (2)	C4^i^—C4—H4	119.8
C3—C2—C2^i^	119.96 (13)	O1—C5—C6	107.3 (3)
C3—C2—C1	120.26 (18)	O1—C5—H5A	110.3
C2^i^—C2—C1	119.77 (10)	C6—C5—H5A	110.3
C4—C3—C2	119.6 (2)	O1—C5—H5B	110.3
C4—C3—H3	120.2	C6—C5—H5B	110.3
C2—C3—H3	120.2	H5A—C5—H5B	108.5
C3—C4—C4^i^	120.43 (14)	C7—C6—C5	174.6 (7)
C3—C4—O1	110.0 (2)	C6—C7—H7	178 (3)
C4^i^—C4—O1	129.56 (15)		
			
C2^i^—C2—C3—C4	0.6 (4)	C5—O1—C4—C3	5.5 (5)
C1—C2—C3—C4	−178.2 (2)	C5—O1—C4—C4^i^	−173.5 (4)
C2—C3—C4—C4^i^	1.2 (4)	C4—O1—C5—C6	173.1 (3)
C2—C3—C4—O1	−177.9 (2)		

Symmetry code: (i) −*x*, *y*, −*z*.

### Hydrogen-bond geometry (Å, º)

**Table:**

*D*—H···*A*	*D*—H	H···*A*	*D*···*A*	*D*—H···*A*
C3—H3···N1^ii^	0.95	2.62	3.509 (3)	155

Symmetry code: (ii) −*x*+1/2, −*y*+1/2, −*z*+1.
